# Identification of *bap* and *icaA* genes involved in biofilm formation in coagulase negative staphylococci isolated from feline conjunctiva

**DOI:** 10.1007/s11259-014-9615-0

**Published:** 2014-09-02

**Authors:** Katarzyna Płoneczka-Janeczko, Paweł Lis, Karolina Bierowiec, Krzysztof Rypuła, Paweł Chorbiński

**Affiliations:** Department of Epizootiology with Clinic for Birds and Exotic Animals, Faculty of Veterinary Medicine, Wrocław University of Environmental and Life Sciences, Plac Grunwaldzki 45, 50-366 Wrocław, Poland

**Keywords:** Conjunctiva, Coagulase negative staphylococci, Biofilm, *bap*, *icaA*, Cats

## Abstract

*Bap* and *icaA* genes are commonly known to be involved in the biofilm formation. The prevalence of *bap* and *icaA* genes and biofilm formation was determined in conjunctival isolates of coagulase negative staphylococci (CNS) collected from cats. The study was conducted on 90 archival CNS isolates collected from feline conjunctiva obtained from clinically healthy cats and cats with ocular problems. Biofilm formation was examined using the microtiter plate (MTP) method. The prevalence of *icaA* and *bap* genes was determined using polymerase chain reaction (PCR). Genetic profiles of the *bap-*positive isolates were examined using the modified random amplified polymorphic DNA (RAPD) method. Of the 90 CNS isolates investigated, 58.9 % (53/90) were confirmed to form biofilms on a polystyrene plate after 24 h, and the intensity of the biofilm production varied strongly between positive strains. Among the biofilm-producing isolates, 24.5 % (13/53) carried the *icaA* gene and 3.8 % (2/53) carried the *bap* gene. Among the isolates that did not produce biofilms, the *icaA* gene and *bap* gene were detected in 8.1 % (3/37) and 2.7 % (1/37) of isolates, respectively. This is the first report demonstrating that CNS isolated from feline conjunctiva can potentially be a *bap* gene reservoir. Preliminary comparison of the genetic profiles of three *bap*-positive isolates collected from cats showed that each of the isolates has a different genetic background with a high similarity with the human strain of *S. epidermidis*.

## Introduction

Differentiation between colonization and infection is a problem in the field of medicine because the same microorganisms can be isolated from clinically healthy animals and animals with clinical manifestations of different infections. Consequently, there has been an increase in the number of studies that focus on commensal microflora and detailed analysis of bacteria, in terms of the decision regarding the eventual introduction of antimicrobial therapy. Researchers have focused on whether commensal microorganisms can exhibit specific invasive traits and properties, which will decide their enhanced virulence (Honda et al. 2010; Murphy et al. 2011)

Microbial biofilm formation is commonly accepted as one of the mechanisms of bacterial virulence as well as resistance (Cucarella et al. 2001; Mah and O’Tolle 2001). The first analysis of microbial cell immobilization in the matrix of extracellular polymers acting as an independent functioning ecosystem that is homeostatically regulated, as biofilms are defined, date back to the 1940s (Percival and Knottenbelt 2011). In recent years, research on the development and presence of biofilms has focused on its implications for the treatment and control of isolated bacteria (Palmer and White 1997). Due to the use of new imaging technologies such as episcopic differential interference contrast microscopy combined with epifluorescence (EDIC/EF) and laser scanning confocal microscopy (LSCM), better visualization of biofilm structure has been achieved (Percival and Knottenbelt 2011)

Various extracellular substances, e.g., extracellular polysaccharides and biofilm-associated proteins, enable bacteria to form biofilms. Production of these components is dependent on the presence of biofilm-essential genes such as the *ica* operon or *bap* gene (Percival and Knottenbelt 2011). *Bap* is a relatively recently identified gene that has been described to encode the surface protein involved in biofilm formation (Valle et al. 2012). The prevalence of the *bap* gene seems to be quite low and ranges between 5 and 10 % in staphylococci isolated from animals with mastitis (Cucarella et al. 2001). In human clinical isolates of *Staphylococcus aureus,* the presence of *bap* gene was not shown (Cucarella et al. 2001). Alternatively, approximately 91 % of human clinical isolates of *Acinetobacter baumanii*, a causative agent of a hospital infection resistant to antimicrobial therapy, were positive for the *bap* gene (Goh et al. 2013). In *bap*-positive isolates of *Staphylococcus aureus*, the described gene was localized within the putative composite transposon inserted in the mobile staphylococcal pathogenicity island SaPIbov2 (Staphylococcal pathogenicity island bovine 2) (Tormo et al. 2005). The *ica* operon (*icaABCD*), with the *icaA* gene (intercellular adhesion gene) responsible for polysaccharide intercellular adhesin poly-*N*-succinyl β-1-6 glucosamine (PIA-PNSG) formation, plays an independent role in the biofilm composition and intercellular adhesion (Cucarella et al. 2001; Potter et al. 2009). In human medicine, up to 80–90 % of clinical isolates of *S. epidermidis* and even one-third of saprophytic strains have been shown to carry the *ica* gene (Møretrø et al. 2003). Biofilms are implicated in approximately 80 % of all infections (urinary tract infections, middle-ear infections, dental plaque, gingivitis or wounds infections), which also are caused by CNS that in the past have long been regarded as nonpathogenic (Percival and Knottenbelt 2011; O’Gara and Humphreys 2001). However, some properties of CNS, such as biofilm production, suggest that these bacteria may play a significant role in the pathogenesis of some infections (hospital-acquired infections, central nervous system shunt infections, endocarditis, urinary tract infections and endophthalmitis) (Huebner and Goldmann 1999) CNS, especially *Staphylococcus simulans*, belong to the resident flora of the feline skin (Scott et al. 2001) The prevalence of CNS isolated from the cat population appears to be related to the clinical status of the animal. In studies carried out on cats in Grenada, CNS have been found in samples obtained from urinary tract infections, pyogenic lesions, and otitis externa cases (Hariharan et al. 2009). In addition, the results of a study among a healthy feral cat population showed a high frequency of *Staphylococcus felis* and *S. simulans* isolation. Bacteria from both species were frequently isolated from feline vagina, ears and eyes (Hariharan et al. 2011). Similarly, Cox et al. (1985) have described *S. simulans* in clinically healthy cats and regarded it to be the most frequently isolated CNS in the examined population (Cox et al. 1985). Moreover, CNS are the predominant bacterial species among the conjunctiva of all companion animal species (Gellat et al. 2013). In clinically normal cats studied by Espinola and Lilenbaum, CNS were detected on the surface of the palpebral conjunctiva, third eyelid, and eyelid margin (Espinola and Lilenbaum 1996). There are no data from veterinary ophthalmology regarding the correlation between some properties of CNS like biofilm production within the conjunctiva and the clinical manifestation of ocular diseases, however, it has been shown that CNS may be implicated in ulcerative keratitis cases in a variety of species, including humans (Lin and Petersen-Jones 2007).

This work presents molecular evidence of *icaA* and *bap* genes, which are involved in biofilm production, in conjunctival isolates of CNS in cats and quantification of the biofilm using the microtiter plate (MTP) method *in vitro*.

## Materials and methods

A total of 90 archival isolates of CNS (bacterial glycerol stocks in 15 % sterile glycerol, stored at −80 °C) collected at the Department of Epizootiology, Faculty of Veterinary Medicine, Wrocław University of Environmental and Life Sciences, were analyzed in this study. In total, 90 isolates of CNS were taken from 90 cats. Thirty-seven CNS isolates were derived from feline conjunctival swabs taken from cats with ocular problems by veterinarians in private veterinary practices. In most cases serous to muco-purulent ocular discharge, oedema and/or hyperemia of conjunctiva were the reasons of examination (described in referrals) and a problem was recognized clinically as conjunctivitis. It is unknown whether the infection was caused by *Chlamydophila felis*, *Mycoplasma felis*, or feline herpes virus (no commissions for laboratory diagnostic). Fifty-three CNS isolates were obtained from clinically healthy animals, without ocular problems. Conjunctival swabs were collected with owner’s consent during visits (vaccination, anti-worming treatment, other laboratory tests) in the Veterinary Practice at the Department of Epizootiology. Identification of the CNS strains (genus level) was performed in the Veterinary Diagnostic Laboratory “Epi-vet” of the Department of Epizootiology by culturing on the BD Mannitol Salt Agar, observing colony characteristics, Gram staining, hemolytic properties on blood agar and resistance to novobiocin. Additionally detection of enzyme production (coagulase tube test) was performed.

Archival strains of bacteria were directly inoculated on Chapman medium and incubated for 24 h at 37 °C. The obtained subcultures (singles colonies) were used for the DNA isolation experiment (Genomic Mini AX Bacteria Spin, A&A Biotechnology, Poland) and the biofilm formation test using the MTP method (Los et al. 2010). The CNS subcultures (single colonies) were suspended in tryptic soy broth (TSB) (Graso, Poland) and cultured for 24 h at 37 °C. Biofilm formation was examined using MTP procedures in a 96-well plate. After an overnight culture in TSB, the culture was diluted 1:100 in TSB, and 200 μl of the suspension was added into three wells. The plate was incubated for 24 h at 37 °C and then washed two times with 200 μl of sterile phosphate-buffered saline. Next, the plate was air-dried for 2 h in an inverted position before it was stained with 200 μl of 0.1 % crystal violet for 10 min. Two biofilm measurement procedures described by Jain and Darwish were tested, but in order to standardize the measurements (possible biofilms diversity), the protocol that dissolved the stain first was selected (Darwish and Asfour 2013; Jain and Agarwal 2009). After the next washing (as described above), the stain bound to bacteria was dissolved by adding 250 μl of 95 % ethanol, and the absorbance was determined at 570 nm on an enzyme-linked immunosorbent assay plate reader (μQuant). The positive controls included two strains with the capacity to form a biofilm: *Staphylococcus epidermidis* PCM 2532 (ATCC 35984) (PAN, Wrocław) and *S. simulans* ATCC 1362, shared by J. R. Penades. As a negative control, sterile TSB was used as proposed by Darwish and Asfour (2013). The MTP procedure was performed three times, in triplicate for each CNS archival strain.

Biofilm-formation capability was considered positive at a cut-off level 0.324, which was established by the mean for the negative control (culture medium, 0.244) plus two standard deviations (0.04). For the positive biofilm formers, the classification criteria were established as follows:

- weak biofilm formers:
  0.324 < A_570_ < 0.648 (2 × negative control)
- medium-positive biofilm formers:
  0.648 < A_570_ < 1.296 (4 × negative control)
- strong biofilm formers:
  A_570_ > 1.296 (4 × negative control)

The presence of the two biofilm-related genes, *icaA* and *bap*, was determined by duplex polymerase chain reaction (PCR), using the modified methods that has been widely studied (Tormo et al. 2005; Hou et al. 2012). The primer sequences used for amplification of *icaA* and *bap* of *Staphylococcus* spp. were as follows:

- *icaA*F: 5′-TCTCTTGCAGGAGCAATCAA-3′
- *icaA*R: 5′-TCAGGCACTAACATCCAGCA-3′
- *bap*F: 5′-CCCTATATCGAAGGTGTAGAATTGCAC-3′
- *bap*R: 5′-GCTGTTGAAGTTAATACTGTACCTGC-3′

The reaction volume was 25 μl, containing: 0.5 μM *icaA*F, 0.5 μM *icaA*R, 0.3 μM *bap*F, 0.3 μM *bap*R, 0.3 mM dNTP, 2.5 μl of DreamTaq Green buffer, 1.5 U of DreamTaq DNA polymerase (Thermo Scientific, Lithuania), and 2 μl of matrix DNA. Amplification was performed as follows: 95 °C for 3 min; 40 cycles of 95 °C for 30 s, 52 °C for 30 s, and 72 °C for 1 min; and 72 °C for 5 min. Electrophoresis was performed on 2 % agarose gel with Midori Green (NIPPON Genetics EUROPE GmbH).

The amplified product was purified using a DNA extraction and purification kit (Fermentas International Inc., Canada). Sequencing reactions of randomly selected *icaA*-positive (*n* = 3) and all *bap*-positive (*n* = 3) PCR products were performed by Genomed (Warsaw, Poland). The sequences were deposited in GenBank (KF972122–KF972127).

The nucleotide sequences of four CNS isolates collected from feline conjunctiva (PJLB-1, PJLB-3, and PJLB-4 for *icaA* and PJLB-1, PJLB-2, and PJLB-3 for *bap*) and sequences obtained from GenBank were aligned by ClustalW software and were analyzed using the Molecular Evolutionary Genetics Analysis (MEGA6) program (Tamura et al. 2004; Tamura et al. 2012). The GenBank database accession numbers of the pathogens used for comparison in this study are as follows:

- *icaA*: gi/111143321/gb/DQ836165.1/*Staph. epidermidis* strain YT-118
  gi/111143324/gb/DQ836166.1/*Staph. epidermidis* strain YT-82
  gi/111143335/gb/DQ836167.1/*Staph. epidermidis* strain YT-55
  gi/111610631/gb/DQ846811.1/*Staph. epidermidis* strain YT-50
  gi/111610633/gb/DQ846812.1/*Staph. epidermidis* strain YT 169-a
  gi/223955852/gb/FJ472951.2/*Staph. haemolyticus strain* 25-59
  gi/402696806/gb/JX298872.1/*Staph. aureus* strain KVAFSU-133
- *bap*: gi/63034432/gb/DQ008306.1/*Staph. epidermidis*
  gi/406047572/gb/JX403946.1/ *Staph. aureus* strain KVAFSU-50
  gi/2624021112/gb/EU011246.2/*Staph. haemolyticus* strain Hp-69
  gi/444329455/gb/HQ170520.3/*Staph. simulans bv. staphylolyticus* strain NNRL 2628
  gi/63034434/gb/DQ008307/ *Staph. hyicus*
  gi/63034428/gb/DQ008304.1/*Staphylococcus xylosus*

Taking into account that in the field of veterinary medicine the *bap* gene involved in biofilm formation has been found only in a small proportion of examined bacteria, the genetic relationship between all confirmed *bap*-positive isolates was established using the modified random amplified polymorphic DNA (RAPD) method described by van Belkum et al., with the primer 1204 (5′-ATGTAAGCTCCTGGGGATTCAC-3′) (van Belkum et al. 1993). The PCR volume was 25 μl, containing 1.6 μM 1204 primer, 1.25 mM dNTP, 2.5 μl of DreamTaq Green buffer, 2 U of DreamTaq DNA polymerase (Thermo Scientific, Lithuania), 1 μl of matrix DNA, and 18.2 μl of milliQ water. The PCR conditions were as follows: 37 cycles of 95 °C for 30 s, 40 °C for 30 s, and 72 °C for 30 s with ramping of 0.2 °C/s in each step. Electrophoresis was performed on 3 % agarose gel with Midori Green (NIPPON Genetics EUROPE GmbH).

A *P* value < 0.05 was considered to indicate a statistically significant association. Statistical analysis of the data in selected groups was accomplished using Fischer’s test and the Student’s t-test.

Ethics Statement. This study was reviewed and approved by the Institutional Animal Care and Use Committee (Local Ethics Commission for Experiments on Animals in Wroclaw, Wroclaw University of Environmental and Life Sciences). Feline conjunctival swabs were taken from cats with ocular problems as well as clinically healthy cats by veterinarians in veterinary practices. The swabs were collected with owner’s consent during visits (vaccination, anti-worming treatment, other laboratory tests) with minimized distress.

## Results

Of the 90 conjunctival CNS isolates investigated, 58.9 % (53/90) were confirmed to form biofilm on the polystyrene plate after 24 h. The percentage of biofilm-positive strains in cats without ocular problems and in cats with clinical manifestation of eyes disease amounted to 54.7 % (29/53) and 64.9 % (24/37), respectively (*p* = 0.38; Fisher’s exact test).

In total, the mean absorbance (A_570_) for all examined CNS strains was measured to be 0.397; and for biofilm-positive strains, it was 0.510. The intensity of the biofilm production varied strongly between positive strains. Of the 53 positive strains examined, 84.9 % (45/53) were confirmed to be weak biofilm formers and 13.2 % (7/53) were medium biofilm formers. Only 1.8 % (1/53) of all positive strains showed a strong capacity to produce biofilm.

The absorbance measurement results (A_570_) for cats with ocular problems and clinically healthy cats with unchanged conjunctiva are given in Table 1. The cats with ocular problems showed higher percentage of medium (13,51 %, 5/37) and strong (2,7 %, 1/37) biofilm-forming CNS isolates than healthy animals (3,77 %, 2/53 and 0 %, 0/53); however, the obtained results were not statistically significant (*p* = 0.06, Fisher’s exact test). Although the mean absorbance for the biofilm-positive strains only was greater (0.560 ± 0.264) in cats with ocular problems than in healthy ones (0.468 ± 0.135) (*p* = 0.10; Student’s t-test) and the mean absorbance calculated for the strong and medium biofilm formers in cats with ocular problems was greater (0.933 ± 0.274) than in healthy ones (0.847 ± 0.205) (*p* = 0.09, Student’s t-test), the differences were not statistically significant. In addition, the ability to form a strong biofilm was confirmed for ill animals only.

**Table 1:** The biofilm production (MTP method, Absorbance A_570_) and presence of *icaA* and *bap* genes in coagulase negative staphylococci (CNS) isolated from feline conjunctiva. Numbers from 1 to 37: cats with ocular problems; numbers from 38 to 90: clinically healthy animals, with unchanged conjunctiva

No of sample	Mean absorbance A_570_	negative BF *	Weak BF*	Medium BF*	Strong BF*	*icaA*	*bap*
Cats with ocular problems							
1.	0,757			+		neg	neg
2.	0,803			+		neg	neg
3.	0,478		+			neg	neg
4.	0,787			+		neg	neg
5.	0,449		+			neg	neg
6.	0,417		+			neg	neg
7.	0,293	+				neg	neg
8.	0,393		+			neg	neg
9.	0,423		+			neg	neg
10.	0,302	+				neg	neg
11.	0,224	+				neg	neg
12.	0,238	+				neg	neg
13.	0,166	+				neg	neg
14.	0,691			+		neg	neg
15.	1,329				+	neg	neg
16.	1,236			+		neg	neg
17.	0,513		+			neg	neg
18.	0,478		+			neg	neg
19.	0,448		+			neg	neg
20.	0,337		+			neg	neg
21.	0,326		+			neg	neg
22.	0,419		+			neg	neg
23.	0,252	+				neg	neg
24.	0,422		+			**POS**	**POS**
25.	0,192	+				neg	neg
26.	0,518		+			neg	neg
27.	0,236	+				neg	neg
28.	0,330		+			neg	neg
29.	0,553		+			neg	neg
30.	0,646		+			neg	neg
31.	0,131	+				neg	neg
32.	0,277	+				neg	neg
33.	0,216	+				neg	neg
34.	0,201	+				neg	neg
35.	0,354		+			neg	neg
36.	0,218	+				neg	neg
37.	0,354		+			POS	neg
ᅟ							
Healthy cats							
38.	0,578		+			neg	neg
39.	0,328		+			neg	neg
40.	0,551		+			neg	**POS**
41.	0,112	+				neg	neg
42.	0,204	+				neg	neg
43.	0,369		+			POS	neg
44.	0,271	+				neg	neg
45.	0,238	+				neg	neg
46.	0,469		+			neg	neg
47.	0,349		+			POS	neg
48.	0,361		+			neg	neg
49.	0,485		+			neg	neg
50.	0,418		+			neg	neg
51.	0,407		+			neg	neg
52.	0,508		+			POS	neg
53.	0,486		+			neg	neg
54.	0,431		+			POS	neg
55.	0,546		+			neg	neg
56.	0,323	+				POS	neg
57.	0,287	+				**POS**	**POS**
58.	0,533		+			POS	neg
59.	0,461		+			neg	neg
60.	0,518		+			**POS**	neg
61.	0,407		+			POS	neg
62.	0,129	+				neg	neg
63.	0,136	+				neg	neg
64.	0,240	+				neg	neg
65.	0,321	+				neg	neg
66.	0,130	+				neg	neg
67.	0,135	+				neg	neg
68.	0,420		+			neg	neg
69.	0,290	+				neg	neg
70.	0,352		+			POS	neg
71.	0,562		+			POS	neg
72.	0,519		+			neg	neg
73.	0,275	+				neg	neg
74.	0,290	+				neg	neg
75.	0,384		+			neg	neg
76.	0,259	+				neg	neg
77.	0,280	+				neg	neg
78.	0,294	+				neg	neg
79.	0,399		+			neg	neg
80.	0,297	+				neg	neg
81.	0,334		+			neg	neg
82.	0,317	+				neg	neg
83.	0,992			+		POS	neg
84.	0,225	+				neg	neg
85.	0,242	+				POS	neg
86.	0,245	+				neg	neg
87.	0,356		+			neg	neg
88.	0,273	+				neg	neg
89.	0,361		+			POS	neg
90.	0,702			+		neg	neg

*BF biofilm formers

Entries in bold indicates the sequenced isolates (*icaA* and *bap*)

Among the biofilm-producing isolates (*n* = 53), 13 (24.5 %) were carriers of the *icaA* gene and 2 (3.8 %) were carriers of the *bap* gene. Among the isolates that did not produce a biofilm (*n* = 37), the *icaA* gene and *bap* gene were detected in 3 (8.1 %) and 1 (2.7 %) CNS strains, respectively. There was a remarkable difference between the prevalence of the *icaA* gene (*p* = 0.01*, Fisher’s test) in cats with unchanged conjunctiva and in cats with ocular problems. However, there was not a statistically significant difference (*p* = 1) in the prevalence of the *bap* gene between these two populations. Table 1 presents a detailed distribution of the *icaA* and *bap* genes.

**Fig. 1:**
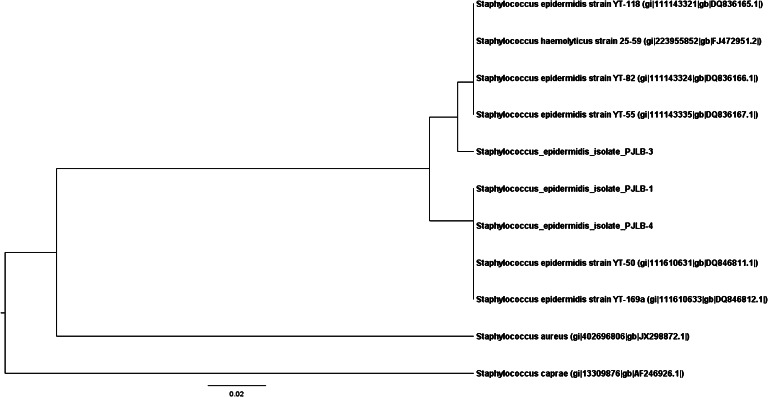
Dendrogram representing the *icaA* gene diversity in conjunctival CNS isolates collected from cats in comparison to other sequences obtained from GenBank. Analyzed strains were recognized as *S. epidermidis*. The similarity tree was inferred using the unweighted pair group method with the arithmetic mean (UPGMA). The optimal tree with the sum of branch length = 0.30669999 is shown. The tree is drawn to scale, with branch lengths in the same units as those of the evolutionary distances used to infer the phylogenetic tree. The evolutionary distances were computed using the maximum composite likelihood method and are in the units of the number of base substitutions per site (Tamura et al. 2004). The analysis involved ten nucleotide sequences. Codon positions included were 1st + 2nd + 3rd + Noncoding. All ambiguous positions were removed for each sequence pair. There were a total of 189 positions in the final dataset. Evolutionary analyses were conducted using MEGA6 software (Tamura et al. 2012)

**Fig. 2:**
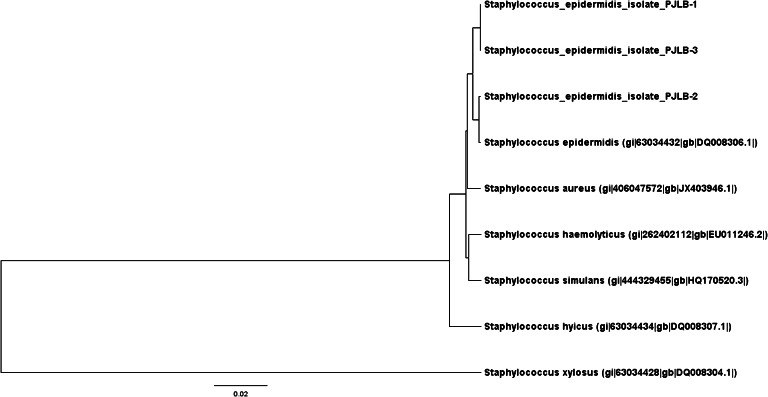
Dendrogram representing the *bap* gene diversity in conjunctival CNS isolates collected from cats in comparison to other sequences obtained from GenBank. Analyzed strains were recognized as *S. epidermidis*. The similarity tree was inferred using the UPGMA method. The optimal tree with the sum of branch length = 0.38994523 is shown. The tree is drawn to scale, with branch lengths in the same units as those of the evolutionary distances used to infer the phylogenetic tree. The evolutionary distances were computed using the maximum composite likelihood method and are in the units of the number of base substitutions per site (Tamura et al. 2004). The analysis involved nine nucleotide sequences. Codon positions included were 1st + 2nd + 3rd + Noncoding. All ambiguous positions were removed for each sequence pair. There were a total of 900 positions in the final dataset. Evolutionary analyses were conducted using MEGA6 software (Tamura et al. 2012)

Figures 1 and 2 show the independent phylogenetic analysis of the *icaA* and *bap* genes confirmed in the CNS strains. Sequencing was performed for four selected CNS strains (No 24, 40, 57, 60), and all double-positive isolates (*icaA* and *bap*) were included. Taking into account the *icaA* gene, comparison of the results obtained by the direct sequencing of three independently collected isolates showed that the sequences of two of them (PJLB-1 and PJLB-4) were identical to *S. epidermidis* YT-50 and YT-169.a. PJLB-3 was different from these two strains and was highly similar to *S. epidermidis* strains YT-55, YT-82, and YT-118, and *Staphylococcus haemolyticus* strain 25–59. Sequence analysis of the *bap* gene showed that strain PJLB-2 was identical to *S. epidermidis* gi/63034432, described by Tormo et al. (2005), and that PJLB-1 and PJLB-3 share high similarity with the isolates described above.

**Fig. 3:**
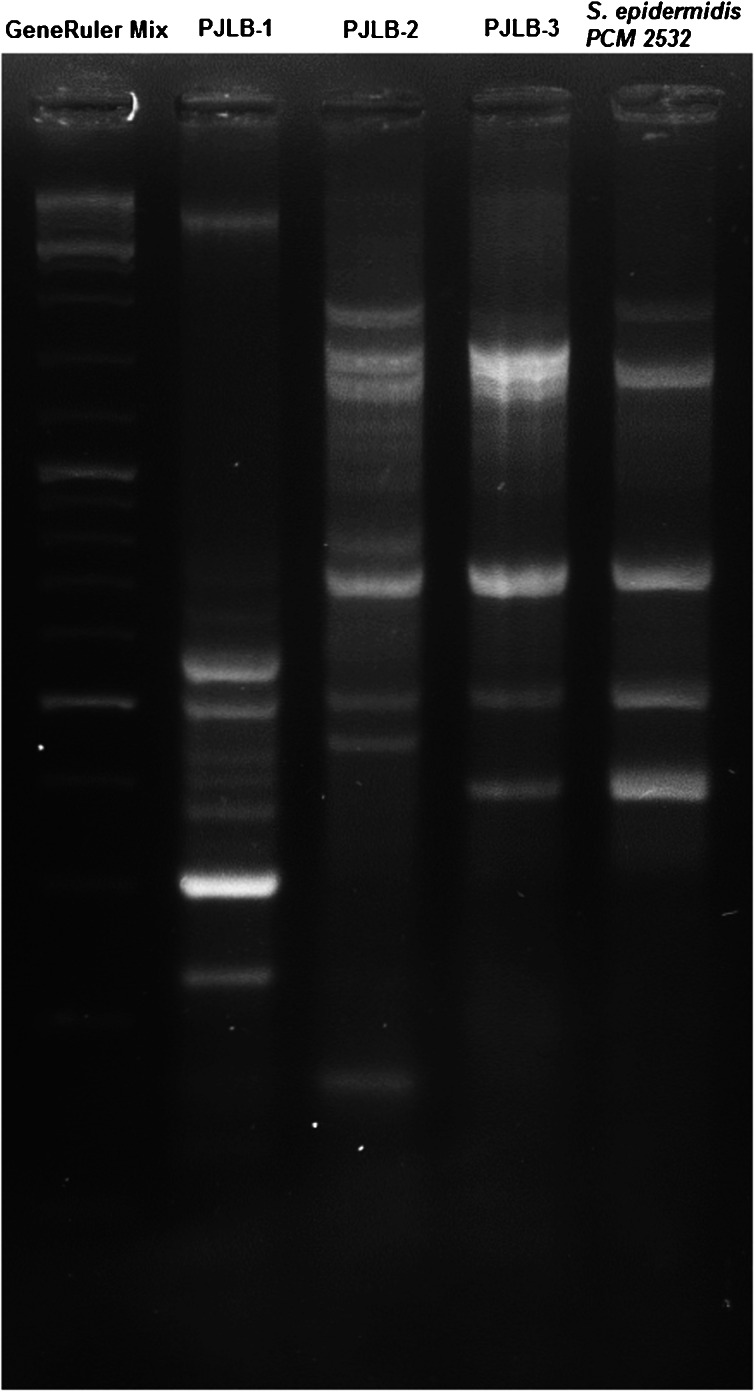
Genetic profiles of the three *bap-*positive isolates of CNS collected from cats and a control strain (PCM 2532) based on the modified RAPD method described by van Belkum et al. (1993)

Figure 3 presents a preliminary comparison of the genetic profiles of the three *bap*-positive isolates of CNS collected from cats, based on the modified RAPD method described by van Belkum et al. (van Belkum et al. 1993). The band pattern obtained in the analysis showed that each of the isolates has a different genetic background and that one of them is highly similar to the human strain of *S. epidermidis* ATCC PCM (PAN, Wrocław).

## Discussion

The results of this study provide clinical significance of CNS and their properties in the fields of human medicine as well as veterinary medicine and food hygiene. CNS have attracted increasing interest as they have been isolated from mastitis in dairy animals, they are potential zoonotic pathogens, and they have the capability to produce enterotoxins in food (El-Jakee et al. 2013; Davis et al. 2013; Podkowik et al. 2013). Another factor that should be taken into account is that CNS may produce biofilms. In the natural environment, this fact may change the potential of the described microorganisms to cause an infection. Biofilm-related genes such as *icaA* or *bap* are involved in cell aggregation and accumulation of the components of the biofilm. The observed differences between the prevalence of the *ica* gene have given rise to discrimination between virulent and avirulent strains (Percival and Knottenbelt 2011).

Biofilm formation is well-documented in staphylococci. It has been studied in humans with ocular infection. Murugan et al. have isolated multi-drug resistant *Staphylococcus* spp. from clinical cases of conjunctivitis and have confirmed that approximately 90 % of them can produce biofilms (Murugan et al. 2010). Biofilms are also implicated in the pathogenesis of acute conjunctivitis related to punctal plugs (Yokoi et al. 2000). In a study by Hou et al. (2012), 28.1 % of investigated CNS bacterial strains were found to be positive using the MTP test and 40.63 % were classified as *icaA* gene carriers. The authors of the mentioned study have suggested that the observed *in vitro* biofilm production may correspond to the biofilm formation within the eye. In addition, Suzuki et al. have described a high prevalence of biofilm-forming *S. epidermidis* with the *icaA* gene (60 %) collected from the conjunctival sac in humans (Suzuki et al. 2005).

In our research, we measured *in vitro* biofilm formation in the setting of veterinary ophthalmology. It is dependent on several genetic determinants, regulators and environmental conditions (Percival and Knottenbelt 2011). Even though the surface of the feline conjunctiva is a difficult place to form biofilms due to tear flow, antibacterial components of the tear film, and cat’s behavior, the ability to produce biofilms *in vitro* was confirmed in a high percentage of examined CNS isolates. The prevalence of biofilm-forming CNS isolates was higher than the prevalence of the two examined genes *icaA* and *bap*. Under various environmental conditions, there are different genetic requirements for biofilm production (Pratt and Kolter 1999). In our research, the biofilm formation properties after 24 h were determined using the MTP test and the presence of chosen genes involved in biofilm formation was examined in molecular studies. The biofilm formation assay in our study was performed only at one set of conditions and far from the natural, *in vivo* conditions. On the other hand, the creation of experimental biofilm-forming conditions mimicking feline conjunctiva, with lacrimal secretions and inhibitory compounds and especially with behavioral factors (scratching, rubbing) exceed the practical possibilities of this research.

The biofilm formation ability among CNS isolates appears to be unrelated to the clinical stage of the conjunctiva; these results in cats contrast with the results obtained in human medicine (Hou et al. 2012; Murugan et al. 2010). However, it should be noted that the feline conjunctivitis is usually caused by primary infection with viruses (FHV-1), *Mycoplasma* spp., or *Chlamydophila* spp. The ability to rule out the abovementioned pathogens limits our conclusions and in the light of obtained results, CNS may be considered only as pathogens that may complicate a primary infection. Acquaintance with the full virological and bacteriological status of feline conjunctiva would allow the determination of whether CNS (and its biofilm formation) may be responsible for ocular problems.

Taking into account that several other genes may be involved in biofilm production, systematic analysis of these genes in conjunctival CNS in cats is needed to determine a correlation with these properties. Since the surface of conjunctiva, by nature, is not optimal place to form biofilm, further research should investigate the adhesion properties of the strains. It has been reported that the *ica* genes are present in CNS from different sources and hosts, whereas the original research describing *bap* suggest that this gene was uniquely present in animals (Li et al. 2012; Szweda et al. 2012). Vautor et al. did not identify the *bap* gene among 262 isolates of *S. aureus* collected from various animal species but cats or dogs were not included in the study (Vautor et al. 2008). In addition, Szweda et al. (2012) examined *S. aureus* strains (*n* = 132) isolated from cows with mastitis, and they found that all strains were also *bap*-negative (Szweda et al. 2012).

In conclusion, this study indicated that the prevalence of biofilm-producing strains among conjunctival isolates of CNS is relatively high, but prevalence alone should not be over-interpreted as critical for the pathogenesis of ocular problems (especially conjunctivitis) associated with CNS in cats. Further studies focusing on the expression of the detected genes of biofilms and prevalence of other genetic determinants is needed, as in the presented study a strain carrying both *bap* and *icaA* genes was isolated, which did not exhibit biofilm formation, possibly due to the variations of expression of the mentioned genes (*bap*) (Thormo et al. 2007). Examined genes that may be involved in the clinical state of conjunctiva in cats and expanding the spectrum of research regarding the genes that may be responsible for these properties may determine the prospective course of veterinary diagnostic investigation in the field of ophthalmology. It would also be interesting to perform a functional study and determine whether it is possible to insert the two examined genes (*icaA* and *bap*) using a plasmid as a vector into biofilm-negative isolates of CNS and estimate whether phenotypic changes (in this case, biofilm formation) follows. It has been shown in *S. aureus* that the presence of a plasmid containing the *bap* gene influences the biofilm formation ability (Cucarella et al. 2001).

Another factor that should be taken into account is whether the eventual transmission of biofilm-forming strains occurs between humans and animals or direct, horizontal transfer of the analyzed genes may exist between the strains (Tormo et al. 2005). Sequencing of the *bap* and *icaA* genes isolated from cats and comparison with sequences from Gene Bank should show that the same isolates of *S. epidermidis* may circulate between cats and humans. Moreover, genetic analysis of *bap*-positive strains with the use of the RAPD method confirmed this hypothesis for one of the tested isolates.

To the best of our knowledge, this is the first report demonstrating the presence of the *bap* gene in conjunctival isolates of CNS collected from cats as well as in *S. epidermidis* isolated from cats. For this reason, this should be considered a risk factor for horizontal transmission of the *bap* gene in the environment.
